# {6-[(2-Anilinoeth­yl)imino­meth­yl]-2-eth­oxyphenolato}(thio­cyanato-κ*N*)­copper(II)

**DOI:** 10.1107/S1600536810010305

**Published:** 2010-03-24

**Authors:** Chen-Yi Wang, Jing-Fen Li, Feng Cao

**Affiliations:** a Department of Chemistry, Huzhou University, Huzhou 313000, People’s Republic of China

## Abstract

In the title complex, [Cu(C_17_H_19_N_2_O_2_)(NCS)], the Cu^II^ atom is chelated by the phenolate O atom, the imine N atom and the amine N atom of the *N*,*N*′,*O*-tridentate 2-eth­oxy-6-[(2-anilino­ethyl)­iminometh­yl]phenolate ligand, and by the N atom of a thio­cyanate anion, forming a distorted CuON_3_ square-planar geometry. The dihedral angle between the aromatic rings of the ligand is 67.9 (4)°. In the crystal, inversion dimers linked by pairs of N—H⋯O hydrogen bonds occur, generating *R*_2_^2^(8) loops.

## Related literature

For background to the structures and properties of copper complexes, see: Collinson & Fenton (1996 ▶); Hossain *et al.* (1996 ▶); Tarafder *et al.* (2002 ▶); Musie *et al.* (2003 ▶); García-Raso *et al.* (2003 ▶); Reddy *et al.* (2000 ▶); Ray *et al.* (2003 ▶); Arnold *et al.* (2003 ▶); Raptopoulou *et al.* (1998 ▶). For related structures, see: Wang *et al.* (2009*a* ▶,*b* ▶); Wang (2009 ▶); Hebbachi & Benali-Cherif (2005 ▶); Butcher *et al.* (2003 ▶); Elmali *et al.* (2000 ▶); Warda *et al.* (1997 ▶).
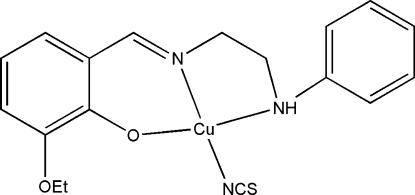


## Experimental

### 

#### Crystal data

[Cu(C_17_H_19_N_2_O_2_)(NCS)]
*M* *_r_* = 404.96
Orthorhombic, 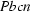

*a* = 13.6786 (5) Å
*b* = 10.4938 (4) Å
*c* = 25.2618 (10) Å
*V* = 3626.1 (2) Å^3^
*Z* = 8
Mo *K*α radiation
μ = 1.34 mm^−1^
*T* = 298 K
0.30 × 0.27 × 0.27 mm

#### Data collection

Bruker SMART CCD diffractometer
Absorption correction: multi-scan (*SADABS*; Sheldrick, 1996 ▶) *T*_min_ = 0.690, *T*_max_ = 0.714
19741 measured reflections
3746 independent reflections
2041 reflections with *I* > 2σ(*I*)
*R*_int_ = 0.069

#### Refinement

*R*[*F* ^2^ > 2σ(*F* ^2^)] = 0.055
*wR*(*F* ^2^) = 0.182
*S* = 1.03
3746 reflections
229 parameters
13 restraints
H atoms treated by a mixture of independent and constrained refinement
Δρ_max_ = 1.25 e Å^−3^
Δρ_min_ = −0.64 e Å^−3^

### 

Data collection: *SMART* (Bruker, 1998 ▶); cell refinement: *SAINT* (Bruker, 1998 ▶); data reduction: *SAINT*; program(s) used to solve structure: *SHELXS97* (Sheldrick, 2008 ▶); program(s) used to refine structure: *SHELXL97* (Sheldrick, 2008 ▶); molecular graphics: *SHELXTL*; software used to prepare material for publication: *SHELXTL*.

## Supplementary Material

Crystal structure: contains datablocks global, I. DOI: 10.1107/S1600536810010305/hb5365sup1.cif

Structure factors: contains datablocks I. DOI: 10.1107/S1600536810010305/hb5365Isup2.hkl

Additional supplementary materials:  crystallographic information; 3D view; checkCIF report

## Figures and Tables

**Table 1:** Selected bond lengths (Å)

Cu1—O1	1.914 (3)
Cu1—N1	1.926 (4)
Cu1—N3	1.941 (4)
Cu1—N2	2.076 (4)

**Table 2:** Hydrogen-bond geometry (Å, °)

*D*—H⋯*A*	*D*—H	H⋯*A*	*D*⋯*A*	*D*—H⋯*A*
N2—H2⋯O1^i^	0.90 (1)	2.07 (3)	2.920 (6)	157 (5)

Symmetry code: (i) 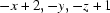.

**Fig. 1.**
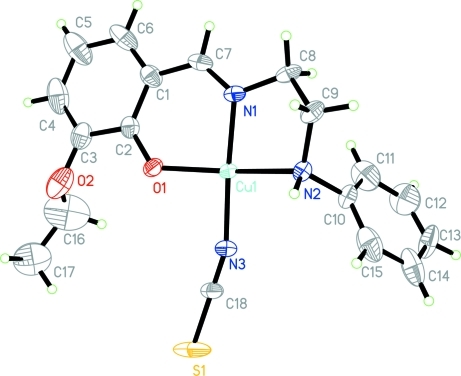
The molecular structure of (I), showing displacement ellipsoids drawn at the 30% probability level.

## supplementary crystallographic information

### Comment

Copper(II) complexes have been received much attention for their versatile biological activities and interesting structures (Collinson & Fenton, 1996; Hossain *et al.*, 1996; Tarafder *et al.*, 2002; Musie *et al.*, 2003; García-Raso *et al.*, 2003). Considerable effort has been made to construct a variety of copper(II) complexes in an attempt to model the physical and chemical behaviour of copper-containing enzymes (Reddy *et al.*, 2000). The peculiarity of copper lies in its ability to form complexes with coordination number four, five, and six (Ray *et al.*, 2003; Arnold *et al.*, 2003; Raptopoulou *et al.*, 1998).

As part of our onging investigations into urease inhibitors (Wang *et al.*, 2009a,b; Wang, 2009), we have synthesized the title compound, (I), a new Cu^II^ complex, and its crystal structure is reported here. The Cu^II^ atom in the complex is chelated by the phenolate O atom, imine N atom, and the amine N atom of 2-ethoxy-6-[(2-phenylaminoethylimino)methyl]phenolate, and by the N atom of a thiocyanate ligand, giving a square planar geometry (Fig. 1). The coordinate bond lengths and angles (Table 1) are typical and are comparable with those observed in other related copper(II) complexes (Hebbachi & Benali-Cherif, 2005; Butcher *et al.*, 2003; Elmali *et al.*, 2000; Warda *et al.*, 1997).

### Experimental

3-Ethoxysalicylaldehyde (1.0 mmol, 166 mg), *N*-phenyl-1,2-diaminoethane (1.0 mmol, 136 mg), ammonium thiocyanate (1.0 mmol, 76 mg), and Cu(CH_3_COO)_2_.H_2_O (1.0 mmol, 200 mg) were dissolved in methanol (80 ml). The mixture was stirred at room temperature for about 1 h to give a blue solution. After keeping the solution in air for a few days, blue blocks of (I) were formed.

### Refinement

H2 was located from a difference Fourier map and refined isotropically, with N—H distance of 0.90 (1) Å. Other H atoms were placed in geometrically idealized positions and constrained to ride on their parent atoms, with C—H distances of 0.93-0.97 Å, and with U_iso_(H) set at 1.2U_eq_(C) and 1.5U_eq_(C17).

### Crystal data

**Table:**

[Cu(C_17_H_19_N_2_O_2_)(NCS)]	*F*(000) = 1672
*M**_r_* = 404.96	*D*_x_ = 1.484 Mg m^−^^3^
Orthorhombic, *P**b**c**n*	Mo *K*α radiation, λ = 0.71073 Å
Hall symbol: -P 2n 2ab	Cell parameters from 2506 reflections
*a* = 13.6786 (5) Å	θ = 2.4–24.9°
*b* = 10.4938 (4) Å	µ = 1.34 mm^−^^1^
*c* = 25.2618 (10) Å	*T* = 298 K
*V* = 3626.1 (2) Å^3^	Block, blue
*Z* = 8	0.30 × 0.27 × 0.27 mm

### Data collection

**Table:**

Bruker SMART CCD diffractometer	3746 independent reflections
Radiation source: fine-focus sealed tube	2041 reflections with *I* > 2σ(*I*)
graphite	*R*_int_ = 0.069
ω scan	θ_max_ = 26.5°, θ_min_ = 1.6°
Absorption correction: multi-scan (*SADABS*; Sheldrick, 1996)	*h* = −17→16
*T*_min_ = 0.690, *T*_max_ = 0.714	*k* = −13→12
19741 measured reflections	*l* = −26→31

### Refinement

**Table:**

Refinement on *F*^2^	Primary atom site location: structure-invariant direct methods
Least-squares matrix: full	Secondary atom site location: difference Fourier map
*R*[*F*^2^ > 2σ(*F*^2^)] = 0.055	Hydrogen site location: inferred from neighbouring sites
*wR*(*F*^2^) = 0.182	H atoms treated by a mixture of independent and constrained refinement
*S* = 1.03	*w* = 1/[σ^2^(*F*_o_^2^) + (0.0843*P*)^2^ + 3.6378*P*] where *P* = (*F*_o_^2^ + 2*F*_c_^2^)/3
3746 reflections	(Δ/σ)_max_ = 0.001
229 parameters	Δρ_max_ = 1.25 e Å^−^^3^
13 restraints	Δρ_min_ = −0.64 e Å^−^^3^

### Special details

**Table:**

Geometry. All esds (except the esd in the dihedral angle between two l.s. planes) are estimated using the full covariance matrix. The cell esds are taken into account individually in the estimation of esds in distances, angles and torsion angles; correlations between esds in cell parameters are only used when they are defined by crystal symmetry. An approximate (isotropic) treatment of cell esds is used for estimating esds involving l.s. planes.
Refinement. Refinement of *F*^2^ against ALL reflections. The weighted *R*-factor wR and goodness of fit *S* are based on *F*^2^, conventional *R*-factors *R* are based on *F*, with *F* set to zero for negative *F*^2^. The threshold expression of *F*^2^ > σ(*F*^2^) is used only for calculating *R*-factors(gt) etc. and is not relevant to the choice of reflections for refinement. *R*-factors based on *F*^2^ are statistically about twice as large as those based on *F*, and *R*- factors based on ALL data will be even larger.

### Fractional atomic coordinates and isotropic or equivalent isotropic displacement parameters (Å^2^)

**Table:**

	*x*	*y*	*z*	*U*_iso_*/*U*_eq_	
Cu1	0.88473 (4)	0.07765 (5)	0.49939 (2)	0.0424 (2)	
O1	0.9189 (3)	0.0617 (3)	0.57256 (13)	0.0498 (9)	
O2	0.9226 (4)	−0.0030 (7)	0.67464 (19)	0.0976 (17)	
S1	0.82168 (12)	−0.35452 (14)	0.52360 (11)	0.1050 (8)	
N1	0.9061 (3)	0.2590 (4)	0.50007 (17)	0.0454 (10)	
N2	0.9049 (3)	0.0938 (4)	0.41821 (16)	0.0468 (10)	
N3	0.8557 (4)	−0.1032 (4)	0.49588 (17)	0.0568 (12)	
C1	0.9081 (4)	0.2849 (6)	0.5943 (2)	0.0625 (15)	
C2	0.9123 (4)	0.1538 (6)	0.6078 (2)	0.0528 (14)	
C3	0.9115 (5)	0.1206 (8)	0.6623 (2)	0.0731 (18)	
C4	0.9057 (6)	0.2158 (12)	0.7003 (3)	0.108 (3)	
H4	0.9043	0.1930	0.7358	0.130*	
C5	0.9020 (7)	0.3417 (12)	0.6870 (4)	0.123 (4)	
H5	0.8990	0.4034	0.7134	0.148*	
C6	0.9027 (5)	0.3772 (8)	0.6348 (4)	0.094 (3)	
H6	0.8995	0.4631	0.6260	0.113*	
C7	0.9110 (4)	0.3275 (5)	0.5412 (3)	0.0585 (15)	
H7	0.9172	0.4149	0.5360	0.070*	
C8	0.9046 (4)	0.3177 (5)	0.4472 (2)	0.0597 (16)	
H8A	0.9436	0.3948	0.4471	0.072*	
H8B	0.8382	0.3397	0.4374	0.072*	
C9	0.9458 (4)	0.2233 (5)	0.4086 (2)	0.0567 (14)	
H9A	0.9303	0.2500	0.3728	0.068*	
H9B	1.0163	0.2207	0.4121	0.068*	
C10	0.8236 (4)	0.0581 (6)	0.3843 (2)	0.0529 (14)	
C11	0.7402 (5)	0.1237 (8)	0.3838 (3)	0.110 (3)	
H11	0.7344	0.1955	0.4052	0.132*	
C12	0.6615 (6)	0.0878 (10)	0.3522 (5)	0.124 (3)	
H12	0.6035	0.1339	0.3538	0.149*	
C13	0.6683 (6)	−0.0091 (11)	0.3208 (3)	0.092 (3)	
H13	0.6174	−0.0291	0.2978	0.110*	
C14	0.7499 (7)	−0.0807 (10)	0.3217 (3)	0.117 (3)	
H14	0.7538	−0.1532	0.3006	0.141*	
C15	0.8299 (6)	−0.0466 (9)	0.3544 (3)	0.105 (3)	
H15	0.8861	−0.0964	0.3551	0.126*	
C16	0.8566 (12)	−0.0681 (15)	0.6821 (7)	0.215 (7)	
H16A	0.8254	−0.0841	0.6483	0.258*	
H16B	0.8102	−0.0210	0.7036	0.258*	
C17	0.8735 (8)	−0.1978 (12)	0.7090 (4)	0.154 (4)	
H17A	0.8550	−0.2650	0.6853	0.232*	
H17B	0.8348	−0.2029	0.7406	0.232*	
H17C	0.9414	−0.2065	0.7180	0.232*	
C18	0.8418 (4)	−0.2070 (5)	0.5072 (2)	0.0523 (13)	
H2	0.952 (3)	0.035 (4)	0.413 (2)	0.080*	

### Atomic displacement parameters (Å^2^)

**Table:**

	*U*^11^	*U*^22^	*U*^33^	*U*^12^	*U*^13^	*U*^23^
Cu1	0.0542 (4)	0.0294 (3)	0.0437 (4)	−0.0002 (2)	−0.0047 (3)	0.0060 (3)
O1	0.058 (2)	0.048 (2)	0.0435 (19)	0.0116 (17)	−0.0025 (16)	0.0050 (16)
O2	0.076 (3)	0.147 (5)	0.069 (3)	0.005 (4)	0.018 (3)	0.042 (3)
S1	0.0524 (9)	0.0341 (8)	0.228 (2)	−0.0038 (7)	−0.0174 (12)	0.0304 (11)
N1	0.042 (2)	0.033 (2)	0.061 (3)	0.0013 (16)	0.000 (2)	0.006 (2)
N2	0.047 (3)	0.051 (3)	0.042 (2)	0.003 (2)	−0.0037 (19)	0.006 (2)
N3	0.074 (3)	0.034 (2)	0.063 (3)	−0.002 (2)	−0.006 (2)	0.004 (2)
C1	0.053 (4)	0.065 (4)	0.069 (4)	0.000 (3)	0.006 (3)	−0.018 (3)
C2	0.047 (3)	0.065 (4)	0.047 (3)	0.001 (3)	0.002 (2)	−0.005 (3)
C3	0.063 (4)	0.102 (5)	0.054 (4)	0.001 (4)	0.004 (3)	0.006 (4)
C4	0.087 (6)	0.182 (10)	0.056 (4)	−0.012 (7)	0.016 (4)	−0.040 (6)
C5	0.115 (8)	0.140 (9)	0.115 (8)	−0.022 (7)	0.028 (6)	−0.067 (8)
C6	0.092 (6)	0.083 (5)	0.106 (6)	−0.011 (4)	0.028 (5)	−0.049 (5)
C7	0.054 (3)	0.036 (3)	0.086 (5)	0.003 (2)	0.005 (3)	−0.007 (3)
C8	0.058 (4)	0.043 (3)	0.078 (4)	0.001 (3)	0.004 (3)	0.028 (3)
C9	0.045 (3)	0.064 (4)	0.061 (3)	0.000 (3)	0.003 (3)	0.022 (3)
C10	0.045 (3)	0.072 (4)	0.042 (3)	−0.003 (3)	−0.003 (2)	0.012 (3)
C11	0.062 (5)	0.122 (7)	0.146 (7)	0.022 (5)	−0.032 (5)	−0.036 (6)
C12	0.068 (6)	0.140 (9)	0.164 (9)	0.010 (5)	−0.048 (6)	−0.012 (7)
C13	0.068 (5)	0.152 (8)	0.056 (4)	−0.039 (6)	−0.019 (4)	0.032 (5)
C14	0.092 (6)	0.166 (9)	0.095 (6)	−0.021 (6)	−0.019 (5)	−0.057 (6)
C15	0.067 (5)	0.140 (8)	0.108 (6)	0.007 (5)	−0.015 (4)	−0.053 (6)
C16	0.199 (10)	0.184 (10)	0.261 (11)	0.002 (8)	0.075 (8)	−0.001 (8)
C17	0.148 (7)	0.157 (8)	0.158 (7)	−0.017 (6)	0.054 (6)	0.036 (6)
C18	0.046 (3)	0.034 (3)	0.077 (4)	0.001 (2)	−0.007 (3)	0.004 (3)

### Geometric parameters (Å, °)

**Table:**

Cu1—O1	1.914 (3)	C7—H7	0.9300
Cu1—N1	1.926 (4)	C8—C9	1.499 (8)
Cu1—N3	1.941 (4)	C8—H8A	0.9700
Cu1—N2	2.076 (4)	C8—H8B	0.9700
O1—C2	1.316 (6)	C9—H9A	0.9700
O2—C16	1.148 (15)	C9—H9B	0.9700
O2—C3	1.342 (9)	C10—C11	1.332 (9)
S1—C18	1.627 (5)	C10—C15	1.336 (9)
N1—C7	1.265 (7)	C11—C12	1.392 (11)
N1—C8	1.470 (6)	C11—H11	0.9300
N2—C10	1.452 (7)	C12—C13	1.294 (12)
N2—C9	1.489 (7)	C12—H12	0.9300
N2—H2	0.901 (10)	C13—C14	1.346 (12)
N3—C18	1.142 (7)	C13—H13	0.9300
C1—C6	1.411 (9)	C14—C15	1.419 (10)
C1—C7	1.414 (8)	C14—H14	0.9300
C1—C2	1.419 (8)	C15—H15	0.9300
C2—C3	1.420 (8)	C16—C17	1.538 (17)
C3—C4	1.388 (11)	C16—H16A	0.9700
C4—C5	1.364 (13)	C16—H16B	0.9700
C4—H4	0.9300	C17—H17A	0.9600
C5—C6	1.371 (13)	C17—H17B	0.9600
C5—H5	0.9300	C17—H17C	0.9600
C6—H6	0.9300		
			
O1—Cu1—N1	92.33 (17)	C9—C8—H8A	110.1
O1—Cu1—N3	90.50 (16)	N1—C8—H8B	110.1
N1—Cu1—N3	176.25 (19)	C9—C8—H8B	110.1
O1—Cu1—N2	158.24 (17)	H8A—C8—H8B	108.4
N1—Cu1—N2	84.73 (18)	N2—C9—C8	110.9 (4)
N3—Cu1—N2	93.54 (17)	N2—C9—H9A	109.5
C2—O1—Cu1	124.9 (3)	C8—C9—H9A	109.5
C16—O2—C3	121.6 (10)	N2—C9—H9B	109.5
C7—N1—C8	120.6 (5)	C8—C9—H9B	109.5
C7—N1—Cu1	125.2 (4)	H9A—C9—H9B	108.1
C8—N1—Cu1	113.8 (3)	C11—C10—C15	118.3 (6)
C10—N2—C9	115.3 (4)	C11—C10—N2	121.9 (6)
C10—N2—Cu1	117.4 (3)	C15—C10—N2	119.7 (6)
C9—N2—Cu1	106.5 (3)	C10—C11—C12	121.9 (8)
C10—N2—H2	107 (4)	C10—C11—H11	119.0
C9—N2—H2	109 (4)	C12—C11—H11	119.0
Cu1—N2—H2	100 (4)	C13—C12—C11	120.6 (9)
C18—N3—Cu1	162.8 (5)	C13—C12—H12	119.7
C6—C1—C7	118.2 (7)	C11—C12—H12	119.7
C6—C1—C2	119.6 (7)	C12—C13—C14	119.2 (7)
C7—C1—C2	122.2 (5)	C12—C13—H13	120.4
O1—C2—C1	123.5 (5)	C14—C13—H13	120.4
O1—C2—C3	118.4 (6)	C13—C14—C15	120.6 (8)
C1—C2—C3	118.1 (6)	C13—C14—H14	119.7
O2—C3—C4	122.7 (7)	C15—C14—H14	119.7
O2—C3—C2	117.5 (6)	C10—C15—C14	119.2 (8)
C4—C3—C2	119.6 (8)	C10—C15—H15	120.4
C5—C4—C3	122.0 (9)	C14—C15—H15	120.4
C5—C4—H4	119.0	O2—C16—C17	118.8 (15)
C3—C4—H4	119.0	O2—C16—H16A	107.6
C4—C5—C6	119.9 (9)	C17—C16—H16A	107.6
C4—C5—H5	120.0	O2—C16—H16B	107.6
C6—C5—H5	120.0	C17—C16—H16B	107.6
C5—C6—C1	120.8 (9)	H16A—C16—H16B	107.1
C5—C6—H6	119.6	C16—C17—H17A	109.5
C1—C6—H6	119.6	C16—C17—H17B	109.5
N1—C7—C1	126.7 (5)	H17A—C17—H17B	109.5
N1—C7—H7	116.7	C16—C17—H17C	109.5
C1—C7—H7	116.7	H17A—C17—H17C	109.5
N1—C8—C9	108.0 (4)	H17B—C17—H17C	109.5
N1—C8—H8A	110.1	N3—C18—S1	179.6 (6)

### Hydrogen-bond geometry (Å, °)

**Table:**

*D*—H···*A*	*D*—H	H···*A*	*D*···*A*	*D*—H···*A*
N2—H2···O1^i^	0.90 (1)	2.07 (3)	2.920 (6)	157 (5)

Symmetry codes: (i) −*x*+2, −*y*, −*z*+1.
